# What Do They Know? Comparing Public Knowledge and Opinions about Rodent Management to the Expectations of Pest Controllers

**DOI:** 10.3390/ani11123429

**Published:** 2021-12-01

**Authors:** Sara A. Burt, Stefan A. Lipman

**Affiliations:** 1 Institute for Risk Assessment Sciences, Faculty of Veterinary Medicine, Utrecht University, 3584 CM Utrecht, The Netherlands; 2 Erasmus School of Health Policy & Management, Erasmus University Rotterdam, 3062 PA Rotterdam, The Netherlands; lipman@eshpm.eur.nl

**Keywords:** rodent, rats, mice, pest control, integrated pest management, IPM, pest controllers, opinion, knowledge

## Abstract

**Simple Summary:**

Control of pests, such as rodents, based on preventive measures and reduced use of non-chemical control is called integrated pest management. Considering the present number of reported rodent infestations, it seems unlikely that the public has much knowledge about rodent pest prevention or integrated pest management. The aim of this study was to find out how much members of the public know about rodents and IPM, and to compare the results with the expectations of pest controllers. In total, 314 members of the public and 86 people working in the pest control sector responded to our online questionnaires. The results show that members of the public have a reasonable level of knowledge regarding preventive measures against rodent control, which are part of integrated pest management. People working in the pest control sector underestimate the public’s knowledge of preventive measures, such as ways of excluding rodents and hygiene measures. Such underestimation may affect their communication with potential clients.

**Abstract:**

Integrated pest management (IPM) involves the control of pests, such as rodents, based on preventive measures and reduced use of chemical control. In view of the number of reported rodent infestations, it appears unlikely that the public has much knowledge about rodents. The objectives of this study were (i) to assess the knowledge and opinions of the public regarding prevention and control of rodent nuisance, and (ii) to assess whether pest controllers have an accurate idea of the knowledge and opinions of the public. The sample contained a total of 314 members of the public and 86 people working in the pest control sector. Responding members of the general public were asked about their knowledge and opinions about IPM in a questionnaire, whereas people working in the pest control sector were asked if they thought the general public had this knowledge and/or opinions. The results show that members of the public have a reasonable level of knowledge regarding preventive measures against rodents, which are part of IPM. People working in the pest control sector underestimate the public’s knowledge of preventive measures, such as perimeter exclusion and hygiene measures. Such underestimation may affect their communication with (potential) clients.

## 1. Introduction

The management of household pests is important to minimize health risks and prevent structural damage to buildings [1,2]. Infestations occur quite frequently; about a third of respondents in a Dutch survey reported that they had had more than one infestation of rodents in the past year [3] and 44% of respondents in an inner-city survey in the UK reported that they currently had mice in the home [4].

**Figure 1:**
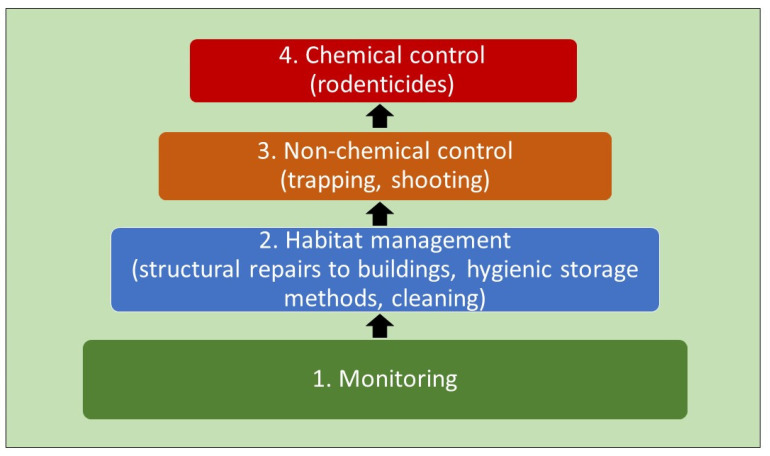
Integrated pest management (IPM) for rodents. Rodent nuisance should be prevented by constant monitoring (1) and the use of exclusion and hygienic practices (2). If culling is necessary, physical methods (3) are preferential to chemical methods (4). Adapted from KMPB, 2021 [6].

In recent years, a sustainable, effective system of pest management has been developed, which combines evaluation of the local ecology and preventive measures with minimal use of hazardous chemicals: integrated pest management (IPM) [5,6]. IPM is almost universally accepted as the best approach because it minimises the effects of pests while also limiting negative effects on public health, animal welfare, and the environment [5,6]. The preventive measures that are part of IPM for rodents include structural measures, such as sealing off entry places in the perimeters of buildings, and hygienic measures, such as storing food and feed in pest-proof containers and cleaning up spills. If these steps are not effective in preventing pest nuisance, non-chemical methods of culling are preferred. Chemical methods (poisons) should be used only as a last resort or if the infestation is so serious that public health is in immediate danger (Figure 1). The reason for the preference of physical methods over chemicals for control is that there is no chance of a spill over of chemicals into the environment or into the food chain of predators [6]. However, it should be noted that physical methods of control, such as the use of traps, can also kill non-target species and scientific research into the effectiveness of these methods is scarce.

Although local authorities deal with pests in public areas, pest management in private homes is, in most countries, the responsibility of the resident. The consistent implementation of structural and hygienic measures when no pest has yet been seen, as prescribed in IPM, involves a proactive approach to pest management that requires some knowledge of the habits and biology of the animals concerned. For example, which entry points would rodents be likely to use? What conditions are attractive to them? Depending on the style and function of the building, technical know-how may also be required. For example, which materials are rodent proof and suitable for sealing off entry points? How small a space can rodents enter through? Only a few studies have been carried out into the knowledge and opinions of the public. However, these studies focused on assessing support for measures of eradication and on which animals are considered pests, rather than on practical knowledge of preventive measures [4,7,8,9,10,11,12]. For example, support for lethal measures of eradication in studies of the general public in various countries ranged from 50% to 91%, with support depending on the species and method used for culling [4,8,9,12,13]. Furthermore, it was shown that the public may have a different assessment of which animals are classed as pests than the authorities generally expect [10], and differences may exist between rodent species, with rats viewed as more negative than mice [7,9]. Importantly, to our knowledge, no studies exist that have assessed the knowledge of and opinions towards the principles of IPM.

One of the reasons so few people take preventive measures could be because they lack the knowledge necessary to be able to effectively protect their homes. When unable to manage pests themselves, the public may need to rely on a pest management company for advice. There is anecdotal evidence in the pest control sector that many people find these preventive measures too much trouble or too expensive, even when they realise action is needed to avoid a potential pest problem [14]. For example, a survey amongst Dutch consumers showed that only about half of people who experienced mouse nuisance stated that they used preventive measures such as sealing the perimeter and storing food out of reach: 43% in 2017 and 50% in 2019 [15].

Hence, there appears to be an important role for pest controllers as a source of information. To effectively communicate the benefits of IPM to residents, pest controllers may need to assess the level of knowledge and opinions of their clients and what their expectations are. In fact, discussing the aim and expectations of the client is one of the first steps in a professional IPM protocol. However, studies comparing expectations of professionals and their customers or patients in other sectors have often shown discrepancies between the expectations of clients and the professional. Such comparisons can highlight useful pointers to improve communication between professionals and clients to optimise the service offered [16,17,18].

We considered that it would therefore be useful to explore the level of knowledge and opinions of members of the public on IPM and compare them to the expectations of pest controllers. Given the high frequency of rodent nuisance experienced by households [3], we hypothesized that the level of knowledge that members of the public have about rodents and IPM is low. Since our study is the first to assess pest controllers’ estimation of the public’s knowledge and opinions, we specified no a priori hypotheses about the accuracy of their estimates. The objectives of this study were (i) to assess the knowledge and opinions of members of the public regarding prevention and control of rodent nuisance, and (ii) to assess whether pest controllers have an accurate idea of the knowledge and opinions of the public.

## 2. Materials and Methods

### 2.1. Sampling Strategy

We recruited two groups of respondents by means of convenience sampling: (i) a sample of the Dutch general public, and (ii) a sample of pest controllers operating in the Netherlands and Belgium. Our sample of the public was recruited via online convenience sampling with responses being collected between February 2019 and December 2019. Recruitment was handled by posting a link to our survey on websites that are visited by homeowners and those searching for information on rodents, i.e., the local health authority (GGD-Regio Utrecht) and the homeowners’ association (Vereniging Eigen Huis). Pest controllers were recruited through multiple channels. First, the lead author recruited pest controllers at a national pest control conference and trade fair on 25 April 2018. Respondents recruited in person (*n* = 10) filled in a paper questionnaire and returned it in person. Second, to increase sample size, both Dutch pest control branch organisations (Nederlandse Vereniging Plaagdiermanagement Bedrijven, NVPB, and Platform Plaagdierbeheersing, PLAN) were contacted with the request to share our survey (programmed in Qualtrics Survey Software) with their members via e-mail. Notifications about the survey were also placed in two Dutch pest control trade journals: Pest Control News and Dierplagen Informatie. Responses (*n* = 76) for this questionnaire were collected between June 2018 and April 2019. Note that, due to the anonymous nature of the survey, there is no guarantee that the 10 pest controllers who filled out the survey in person did not fill out the online survey as well.

### 2.2. Survey Questions

The questions used for measuring knowledge and opinions were adapted from pre- and post-IPM workshop surveys aimed at care staff in a U.S. study [19,20]. English translations of the surveys can be found in the supplementary material (Files S1 and S2). The surveys contained nine propositions related to rodents and pest control (e.g., ‘Rats and mice are parts of Dutch natural fauna’ and ‘Filling up holes in walls will prevent rodents from entering the building’). The general public was asked if these nine propositions were true or not (Yes/No), and how certain they were on a scale from 1 (‘completely unsure’) to 7 (‘completely sure’). Pest controllers, on the other hand, were asked to report if they expected the public to know if these statements were true (Yes/No) and how certain they were the general public knew the veracity of these propositions on the same 7-point scale. The five remaining questions were set up similarly and captured opinions towards pest control carried out by professional companies or municipalities. The public reported whether they held this opinion and how sure they were of their opinion. Again, pest controllers were asked if they expected the general public to hold these opinions and the certainty with which they held this expectation.

Besides measuring knowledge and opinions, we collected a range of professional and personal characteristics. For the general public, data was collected on gender, age group, education level, student status, pet ownership, rodent ownership, type of residence (including build year), area (rural or urban), and how often in the past year respondents suffered from pests in or around the house. For pest controllers, the following data was collected: type of pest control organisation, client types, accreditation, country in which most work is completed, duration of pest control occupation, gender, and age group. All participants provided informed consent and the data were analysed anonymously.

### 2.3. Statistical Analyses

All analyses were performed in R statistical software (version 4.0.5) [21] (data and analysis scripts available on request). Knowledge and opinions of the general public were first summarized. Afterwards, the proportion of responding members of the public that agreed with each statement was compared to the expected agreement predicted by pest controllers through Chi-squared tests. The expected and actual certainty associated with each statement was compared through *t*-tests.

## 3. Results

### 3.1. General Public Demographic Characteristics

Our convenience sample of the general public yielded a total of 314 responses (Table 1). A large majority was female. The highest education level achieved was spread across categories from secondary school, technical college, to higher education. Three quarters of respondents owned pets (about quarter of them included rodents) and a small percentage of farm animals. Almost half of respondents lived in a terraced house and about a quarter in a detached house. The balance between urban and rural location was about 40:60. Slightly more than half of respondents lived in a home built after 1960. About one sixth of respondents had never had a rodent problem in the home, about the same number had rodent problems almost every year, and the rest had had rodent problems several times in the past. Although the survey was launched via university channels, only 12% were a students.

### 3.2. Pest Controller Demographic Characteristics

A total of 86 people working in the pest control sector completed the survey for pest controllers (10 on paper and 76 online) and their demographic characteristics are presented in Table 2. A large majority were male and had worked in this sector for more than 5 years. More than two thirds had an official pest control qualification. About two thirds of respondents were employed by a pest control company and the rest were employed by local government, an advisory bureau, or a company that supplies pest control products. The clients were spread over various sectors (local government, agriculture, private householders, and care institutions) and many respondents had clients in more than one of these sectors.

### 3.3. Public Knowledge and Opinions Regarding Rodent Control

The results of statements on points of factual knowledge and statements reflecting opinions are presented in Table 3.

For all nine statements expressing correct facts on IPM, most respondents from the public were in agreement. Respondents were most certain of the statements that rodents need food, water, and shelter to survive, and that food should be stored in containers with tightly fitting lids. The statement that they were least certain about was that approved rodenticides can be recognised by a registration number on the packaging.

The most agreed upon opinion was ‘If I experienced rodent nuisance, I would contact the local authority’. The level of certainty for this opinion was also high. A large majority of respondents agreed that, if it was necessary to kill rodents, that humane methods should be used and that the use of traps was preferable above poisons. Almost two-thirds stated that they would contact a specialist company if they encountered rodent nuisance. The statement with the lowest level of agreement (just over half of respondents) was ‘If I experience nuisance from rodents I know where I can get advice’.

### 3.4. Expected vs. Actual Knowledge and Opinions about Rodents

Table 3 shows that the public response to only 2 of the 14 statements was accurately predicted by the pest controllers, i.e., the proportion of expected agreement was not statistically different from actual agreement for these two questions. These statements concerned whether the public knew where they could get advice about pest management, and whether the public would enlist the help of a specialist company if they experienced pest nuisance. For the remaining 12 statements, people working in the pest control sector underestimated the proportion of agreement in the general public with the statements reflecting knowledge and opinions (*p* < 0.05) (Table 3).

Generally, the public were more certain of their answers than predicted by pest controllers. Differences in certainty were particularly large for statements regarding everyday methods of preventing rodent nuisance (excluding rodents from buildings, sealing cracks in walls, storing food in sealed containers, removal of rubbish), for which the public was *more* certain than the pest controllers expected. The public was also highly certain that they would contact the local authority if they experienced rodent nuisance, and this certainty was significantly higher than predicted by pest controllers. The public was also significantly *more* certain than the sector expected that lethal pest control methods should be humane. The public was also more certain than expected that rats and mice were part of the natural fauna of the Netherlands and that rodents need food, water, and a resting place in order to survive. The only statement for which the public was significantly *less* certain than the pest controllers had expected, was that government-approved rodenticides could be recognised by the registration number on the label.

## 4. Discussion

Accurate knowledge about IPM may be a prerequisite for members of the public to take sufficient protective measures. The current suboptimal uptake of IPM, as shown by levels of infestations, suggests that the general public may not be sufficiently informed about its necessity and benefits. This study set out to explore the knowledge and opinions of the general public about IPM. Furthermore, we also compared the public’s knowledge and opinions with pest controllers’ expectations about these matters. The latter may be of importance as pest controllers will generally be one of the nearest sources of advice to the public on rodent control.

The percentage of participants from the public that agreed with the nine correct statements on IPM was 70.1–98.7%. This indicates that the majority of respondents can recognise correct statements about preventive measures against rodent nuisance. People working in the pest control sector underestimated the proportion of the public that would agree with these statements. For example, more than 90% of public respondents agreed that excluding rodents from buildings is preferable to using traps or poison, and 87% acknowledged that rodent infestations can be prevented by not leaving food or rubbish out where rodents have access to it. These points were underestimated by the pest controllers, who expected a significantly lower percentage of the public would regard these statements as true. These findings suggest that, although people working in the pest control sector are pessimistic about householders’ willingness to invest in preventative measures [14], there is potential for activating preventive IPM-behaviour in their clients. A U.S. study carried out amongst the directors of childcare homes also revealed a relatively high level of knowledge about IPM topics. There, 75–95% of the 20 directors indicated the correct answers to statements about IPM *prior* to taking part in a workshop about IPM [20].

The pest control sector also strongly underestimated the proportion of the public that stated that humane methods should be used if rodents need to be culled and the proportion that preferred the use of traps over poison in regard to reducing the risk of spreading poison in the environment. It is possible that members of the public have chosen the socially acceptable answers. Anecdotal evidence from the sector indicates that, although people may be in favour of humane methods in principle, if their own home is under threat, they can be less discriminating.

Public respondents were uncertain of how to recognise approved rodenticides, even though most had the answer correct. Pest controllers underestimated the number of people who got the answer right but overestimated their level of certainty. Clearly, there is a need for authorities and the pest control retail sector to provide information on this.

The statement with the highest level of agreement was that the public would contact the local authority if they experienced rodent nuisance. The lowest level of agreement (and a low level of certainty) was for knowing where to get advice on rodent nuisance. These two points taken together suggest that people consider the local authority to be the first port of call, even if they are uncertain whether it is able to advise them. Some people may expect that the council would tackle the problem for them. Our finding that people generally do not know where to get advice on rodents is similar to the results of a study amongst Dutch consumers in 2017 and 2019, which showed that slightly over half did not seek advice before dealing with a rodent problem, and 16% searched for information on the internet [15]. The lack of clarity about where advice on rodent control can be obtained reflects the current situation in the Netherlands—there is no longer a central source of advice or coordination for pest control at national level. 

Almost two thirds of public respondents said they would contact a specialist company if they encountered rodent nuisance and pest controllers estimated a similar proportion and level of certainty. However, these data contrast with the findings of a study amongst Dutch consumers in which only 2–20% stated that they had engaged a specialist pest controller to tackle a rodent problem [15], and a UK study where 15–45% said they had engaged a professional when they experienced rodent nuisance [7].

Whether people act on the knowledge they have depends on a number of factors. Often there is a discrepancy between intentions and actual behaviour, known as the ‘intention behaviour gap’ [22], and a cue is often required before people act on their knowledge or change their behaviour [23]. In the case of rodent control, the cue to action could be seeing a rodent in the house. Previous research has shown that intentions to carry out pest control depends on perceived benefits, perceived barriers, and the severity of the threat [3]. It may be possible for the government or local authorities to provide a motivational cue that would encourage the implementation of appropriate preventive measures, such as launching an awareness campaign. Other researchers have pointed out that raising awareness of the risks of pests can shift risk perception, reduce tolerance levels, and promote preventive behaviours [24,25]. Emphasising the sustainable benefits of IPM, such as reducing rodenticide use and safeguarding biodiversity, may lead to shifts in common practice [26] and making people aware of the effectiveness of preventive environmental management would motivate them to take action [4]. Research in Australia has shown that well-run pest awareness campaigns can significantly raise public awareness [10] and a U.S. study showed that elementary training in IPM can be successful in improving knowledge, raising awareness, and reducing the number of rodent pests in a work situation [27]. Several Dutch municipalities recently updated their policies on pest prevention and have launched information campaigns to prompt the public into better preventive behaviours (Municipality of Utrecht, https://youtu.be/P5SExtJ-80c, last accessed 12 October 2021; Municipality of Amsterdam, https://www.stopderat.nl/deel-en-test-je-kennis/, last accessed 12 October 2021).

## 5. Limitations of the Study

Since this was an online survey, selection bias may have occurred if members of the public who have experienced rodent nuisance in the past were more likely to take part. This may have artificially raised the level of knowledge about rodents. Furthermore, a result of our convenience sampling strategy is that the number of female respondents was approximately four times the number of male respondents. Such unrepresentative sampling may be a source of bias, which could be avoided in future studies using representative sampling. The results reported in the supplementary material (File S3) explore the extent to which this may have biased our results by comparing knowledge and opinions between males and females, as well as between people living in rural and urban areas. Furthermore, due to the online character of the survey, it is also possible that the average age for participating members of the public is lower than it would have been if a personal interview or postal questionnaire had been used.

Some questions posed were hypothetical, since they asked about what people would do if they had a rodent problem. A discrepancy exists between intentions and actual behaviour (Sheeran 2002), so these questions may suffer from hypothetical bias. Another limitation was that the correct answer to all the questions about knowledge was ‘yes’, which may have led respondents to identify the correct answer regardless of their actual knowledge. On balance, this would lead to an overestimation of respondents’ knowledge. Future work may prevent such bias by phrasing some questions negatively, such that the correct answer would vary by question, or by using open answers instead.

The choice for using a certainty scale for answers instead of a ‘don’t know’ option provided more information in the form of a numerical score. However, this may have reduced the validity of some of the answers where a high number of people answered ‘yes’ but indicated low certainty.

## 6. Recommendations for Policy and Future Research

Although 70–90% of respondents in this study agreed with statements on IPM, it is known that many households do suffer rodent nuisance. Authorities could build on the passive knowledge of rodents and IPM that the public has by encouraging the public to act on what is known and by giving ‘cues’ to implement preventive elements of IPM. For example, promotional campaigns, particularly in inner city areas with rodent problems, could trigger IPM behaviours. This can be complemented by supplying clear information as to where information and advice on rodent control can be sought and how to recognise approved rodent control products. A central source of advice and coordination of pest control in the Netherlands would improve the situation. Some local authorities carry out rodent control in homes, some carry out rat control in public areas and only advise on mouse control in the home. Others merely provide a list of pest control companies to contact. Consequently, there is no national uniformity, and the local situation is often unclear. This finding highlights a task for local and national government—to make it clearer where information and advice can be sought.

## 7. Conclusions

This study shows that responding members of the public have a reasonable level of passive knowledge regarding preventive measures against rodent nuisance, which are part of IPM. A key finding is that people working in the pest control sector underestimate the public’s knowledge of preventive measures, such as perimeter exclusion and hygiene measures. The proportion of the public that thinks humane methods should be used for rodent control was also higher than predicted by the sector. As such, our findings suggest that there may be more potential for activating preventive IPM behaviour in their clients than pest controllers expect. These findings may promote awareness of the knowledge level and opinions of the public for the pest control sector and the local authorities and provide a starting point for coordinated promotional activities which could cue people to better implement IPM and reduce rodent nuisance.

## Figures and Tables

**Table 1:** Demographics for respondents from the general public (*n* = 314).

		Number of Participants	Percentage (%)
Gender	Male	58	18.5
	Female	255	81.2
Age (mean/SD)	(43.99/14.23)		
Education	Secondary school	42	13.4
	Technical college	104	33.1
	Vocational training	67	21.3
	University	94	29.9
	Other	7	2.2
Student	Yes	37	11.8
	No	276	87.9
Animal owner	Yes	231	73.6
	No	72	22.9
	Farmer	11	3.5
Pet is rodent	Yes	57	18.2
	No	183	58.3
Type of house	Flat on ground floor	11	3.5
	Flat on upper floor or maisonette	52	16.6
	Terraced	134	42.7
	Semi-detached	31	9.9
	Farm with animals	6	1.9
	Detached house	71	22.6
	Other	9	2.9
Urban or rural area	Village	186	59.2
	Town or city	127	40.4
Year house was built	Before 1960	130	41.4
	1960 or later	173	55.1
	Unknown	10	3.2
Rodent nuisance	Several times per year	60	19.1
	Almost every year	50	15.9
	Several times in recent years	60	19.1
	A couple of times in recent years	95	30.3
	Never	49	15.6

**Table 2:** Demographics for respondents from the pest control sector (*n* = 86).

		Number of Participants *	Percentage (%)
Employer	Pest control company	10	12
	Advisory bureau	4	5
	Supplier of pest control materials	5	6
	Local authority	8	10
	Other	68	81
Clients are	(Local) government	54	64
	Agricultural sector	31	37
	Companies	68	81
	Private citizens	66	79
	Care institutions	50	60
	Other	22	26
In possession of pest control certification	Yes	74	87
	No	11	13
Active in country	Netherlands	82	97
	Belgium	2	2
	Worldwide	1	1
Work experience as pest controller	More than 5 years	65	76
	5 years or less	16	19
	I do not work in the pest control sector	4	5
Gender	Male	78	92
	Female	7	8

* None of the questions were mandatory, so not all respondents answered all questions.

**Table 3:** Results for knowledge and opinions of the general public compared to knowledge and opinions expected by pest controllers. Participants indicated their agreement with the statements (Yes/No) and their degree of certainty (on a scale from 1–7).

	Agreement with Statement		Certainty of Statement	
	General Public (*n* = 314)	Pest Controllers (*n* = 86)	General Public (*n* = 314)	Pest Controllers (*n* = 86)
Knowledge	Number (%)	Number (%)	Mean (SD)	Mean (SD)
Q1 Rats and mice belong to the natural wild fauna of the Netherlands.	298 (94.9%) *	65 (77.4%)	**6.11 (1.24)**	5.53 (1.26)
Q2 Rodents need food, water, and shelter to survive.	**310 (98.7%)**	67 (79.8%)	**6.43 (1.11)**	5.53 (1.35)
Q3 You can prevent rodents entering buildings by sealing off cracks in the walls.	**221 (70.4%)**	34 (40.5%)	5.30 (1.47)	5.26 (1.46)
Q4 Climbing plants growing up against the exterior walls make it easier for rodents to enter buildings.	**250 (79.6%)**	13 (15.5%)	5.32 (1.56)	5.51 (1.48)
Q5 Excluding rodents from buildings is preferential to using traps or poison.	**292 (93.0%)**	12 (14.3%)	**6.1 (1.34)**	5.40 (1.59)
Q6 Food should be stored in containers with tightly fitting lids.	**294 (93.6%)**	37 (44.0%)	**6.44 (1.10)**	5.41 (1.26)
Q7 Rodent infestations can be prevented by not leaving food or rubbish out.	**276 (87.9%)**	49 (58.3%)	**6.03 (1.30)**	5.37 (1.26)
Q8 In serious cases of rodent infestation it may be necessary to eradicate the animals by using traps or poison.	**256 (81.5%)**	78 (92.9%)	5.80 (1.54)	5.91 (1.18)
Q9 A rodenticide is approved for use in the Netherlands if it has a N-number or NL-number on the packaging.	**220 (70.1%)**	11 (13.1%)	**3.01 (1.91)**	5.88 (1.83)
Opinions				
Q1 If I experienced rodent nuisance, I would contact the local authority.	**266 (84.7%)**	34 (40.5%)	**6.11 (1.34)**	5.33 (1.37)
Q2 If it is necessary to kill rodents because they have become a pest, this should be done using humane methods.	**250 (79.6%)**	16 (19.0%)	5.50 (1.68)	5.26 (1.46)
Q3 The use of traps is preferable to the use of poison because with traps there is no risk of spreading poison in the environment.	**195 (62.1%)**	41 (48.8%)	**5.62 (1.66)**	5.20 (1.25)
Q4 If I experience nuisance from rodents I know where I can get advice.	166 (52.9%)	46 (54.8%)	5.46 (1.61)	5.32 (1.12)
Q5 If I experience nuisance from rodents, I will contact a specialist company.	190 (60.5%)	48 (57.1%)	4.97 (1.86)	4.89 (1.49)

* Boldface indicates that these proportions or certainty values differed from what pest controllers expected (*p* < 0.05).

## Data Availability

The anonymised data presented in this study are available on request from the corresponding author. The data are not publicly available as no explicit permission was requested from respondents to share their data publicly.

## Supplementary Materials

The following are available online at https://www.mdpi.com/article/10.3390/ani11123429/s1, File S1: Questionnaire for members of the public (English translation); File S2: Questionnaire for pest controllers (English translation). File S3: Comparisons of results for (i) female vs. male members of the public, and (ii) members of the public living in urban vs. rural areas.

## References

[1] Jahan N.A., Lindsey L.L., Larsen P.A. (2021). The Role of Peridomestic Rodents as Reservoirs for Zoonotic Foodborne Pathogens. Vector Borne Zoonotic Dis..

[2] Meerburg B.G., Singleton G.R., Kijlstra A. (2009). Rodent-borne diseases and their risks for public health. Crit. Rev. Microbiol..

[3] Lipman S.A., Burt S.A. (2017). Self-reported prevalence of pests in Dutch households and the use of the health belief model to explore householders’ intentions to engage in pest control. PLoS ONE.

[4] Marshall P.A., Murphy R.G., Meehan A.P. (1984). Investigating residents’ perceptions of urban rodents in Manchester, UK. Rats and Mice. Their Biology Control.

[5] Centers for Disease Control and Prevention (2006). Integrated Pest Management: Conducting Urban Rodent Surveys.

[6] KPMB (2021). Handboek IPM Knaagdierbeheersing, Versie 2.0, 21 April 2021.

[7] Baker S.E., Maw S.A., Johnson P.J., Macdonald D.W. (2020). Not in My Backyard: Public Perceptions of Wildlife and ‘Pest Control’ in and around UK Homes, and Local Authority ‘Pest Control’. Animals.

[8] Bremner A., Park K. (2007). Public attitudes to the management of invasive non-native species in Scotland. Biol. Conserv..

[9] Farnworth M.J., Watson H., Adams N.J. (2014). Understanding Attitudes Toward the Control of Nonnative Wild and Feral Mammals: Similarities and Differences in the Opinions of the General Public, Animal Protectionists, and Conservationists in New Zealand (Aotearoa). J. Appl. Anim. Welf. Sci..

[10] Fisher N.I., Lee A.J., Cribb J.H.J. (2013). A Scientific Approach to Monitoring Public Perceptions of Scientific Issues. Int. J. Sci. Educ. Part B.

[11] Garba M., Kane M., Gagare S., Kadaoure I., Sidikou R., Rossi J.P., Dobigny G. (2014). Local perception of rodent-associated problems in Sahelian urban areas: A survey in Niamey, Niger. Urban Ecosyst..

[12] Sked S., Abbar S., Cooper R., Corrigan R., Pan X., Ranabhat S., Wang C. (2021). Monitoring and Controlling House Mouse, Mus musculus domesticus, Infestations in Low-Income Multi-Family Dwellings. Animals.

[13] Fraser W. (2001). Introduced Wildlife in New Zealand: A Survey of General Public Views.

[14] Van Gerwen M.A.A.M., Nieuwland J., Van Lith H.A., Meijboom F.L.B. (2020). Dilemmas in the Management of Liminal Rodents-Attitudes of Dutch Pest Controllers. Animals.

[15] Komen C.M.D., Wezenbeek J.M. (2021). Particulier Gebruik van Rodenticiden en Middelen Tegen Groene Aanslag. RIVM-Briefrapport 2020-0072.

[16] Demetriou J.L., Geddes R.F., Jeffery N.D. (2009). Survey of pet owners’ expectations of surgical practice within first opinion veterinary clinics in Great Britain. J. Small Anim. Pract..

[17] Mudiyanse R.M., Weerasinghe G.S.M., Piyasinghe M.K., Jayasundara J.M.H. (2015). Patient’s Expectations during Doctor Patient Communication and Doctors Perception about Patient’s Expectations in a Tertiary Care Unit in Sri Lanka. Arch. Med..

[18] Sladdin I., Ball L., Gillespie B.M., Chaboyer W. (2019). A comparison of patients’ and dietitians’ perceptions of patient-centred care: A cross-sectional survey. Health Expect..

[19] Alkon A., Kalmar E., Leonard V., Flint M.L., Kuo D., Davisdson N., Bradman A. (2012). Development and Evaluation of an Integrated Pest Management Toolkit for Child Care Providers. Early Child. Res. Pract..

[20] Stephens M., Hazard K., Moser D., Cox D., Rose R., Alkon A. (2017). An Integrated Pest Management Intervention Improves Knowledge, Pest Control, and Practices in Family Child Care Homes. Int. J. Environ. Res. Public Health.

[21] R Core Team (2015). R: A Language and Environment for Statistical Computing.

[22] Sheeran P. (2002). Intention—Behavior relations: A conceptual and empirical review. Eur. Rev. Soc. Psychol..

[23] Janz N.K., Becker M.H. (1984). The health belief model: A decade later. Health Educ. Behav..

[24] Aerts C., Revilla M., Duval L., Paaijmans K., Chandrabose J., Cox H., Sicuri E. (2020). Understanding the role of disease knowledge and risk perception in shaping preventive behavior for selected vector-borne diseases in Guyana. PLoS Negl. Trop. Dis..

[25] Schoelitsz B., Poortvliet P.M., Takken W. (2018). Factors driving public tolerance levels and information-seeking behaviour concerning insects in the household environment. Pest Manag. Sci..

[26] Lowe E.C., Latty T., Webb C.E., Whitehouse M.E.A., Saunders M.E. (2019). Engaging urban stakeholders in the sustainable management of arthropod pests. J. Pest Sci..

[27] Alkon A., Nouredini S., Swartz A., Sutherland A.M., Stephens M., Davidson N.A., Rose R. (2016). Integrated Pest Management Intervention in Child Care Centers Improves Knowledge, Pest Control, and Practices. J. Pediatr. Health Care.

