# Supplementary material for: What Do They Know? Comparing Public Knowledge and Opinions about Rodent Management to the Expectations of Pest Controllers
Source: Animals (Basel). 2021 Dec 1;11(12):3429. doi: 10.3390/ani11123429 (PMC8698193; doi:10.3390/ani11123429)
Supplement: Supplementary file 1 [file animals-11-03429-s001.zip › S1_English translation of questions for the Public.pdf]

Questionnaire for members of the general public - *English translation of the statements offered.*

**Please indicate whether you agree with the statements**	Yes No	How certain are you? (1 = very unsure – 7 absolutely certain) 1 2 3 4 5 6 7
1.Rats and mice belong tot he natural fauna of the Netherlands	O O	O O O O O O O
2.Rodents need food, water and a resting place in order to survive	O O	O O O O O O O
3.You can prevent rodents from entering builidings by sealing cracks and holes in the walls.	O O	O O O O O O O
4.Having climbing plants growing up the exterior walls makes it easier for rodents to enter buildings	O O	O O O O O O O
5.Excluding rodents from buildings by preventive technical measures is preferable to using poison	O O	O O O O O O O
6.Food should be stored in containers with sealed lids	O O	O O O O O O O
7.Rodent nuisance can be prevented by making sure that no food or rubbish is left out	O O	O O O O O O O
8.In cases of severe infestation it may be necessary to cull the animals with traps or poison	O O	O O O O O O O
9.Government approved rat and mouse poisons can be identified by a number followed by N or NL on the label	O O	O O O O O O O
**Please indicate whether you agree with these opinions**	Yes No	How certain are you? (1 = very unsure – 7 absolutely certain) 1 2 3 4 5 6 7
10.If it is necessary to kill rodents because they are causing a nuisance, this should be done in a humane way	O O	O O O O O O O
11.Using traps is preferable to using poison because there is less risk of spreading poison in the environment	O O	O O O O O O O
12.If you were to experience rodent nuisance, you know where you can get advice	O O	O O O O O O O
13.If you were to experience rodent nuisance, you would engage a specialist company	O O	O O O O O O O
14.If you were to experience rodent nuisance, you would contact the local authority	O O	O O O O O O O
