# Supplementary material for: What Do They Know? Comparing Public Knowledge and Opinions about Rodent Management to the Expectations of Pest Controllers
Source: Animals (Basel). 2021 Dec 1;11(12):3429. doi: 10.3390/ani11123429 (PMC8698193; doi:10.3390/ani11123429)
Supplement: Supplementary file 1 [file animals-11-03429-s001.zip › S2_English translation of questions for Pest controllers.pdf]

Questionnaire for pest controllers - *English translation of the statements offered.*

**In general, do members of the public know that …**	Yes	No	How certain are you? (1 = very unsure – 7 absolutely certain) 1	2	3	4	5	6	7
1.Rats and mice belong to the natural fauna of the Netherlands	O	O	O	O	O	O	O	O	O
2.Rodents need food, water and a resting place in order to survive	O	O	O	O	O	O	O	O	O
3.You can prevent rodents from entering builidings by sealing cracks and holes in the walls.	O	O	O	O	O	O	O	O	O
4.Having climbing plants growing up the exterior walls makes it easier for rodents to enter buildings	O	O	O	O	O	O	O	O	O
5.Excluding rodents from buildings by preventive technical measures is preferable to using poison	O	O	O	O	O	O	O	O	O
6.Food should be stored in containers with sealed lids	O	O	O	O	O	O	O	O	O
7.Rodent nuisance can be prevented by making sure that no food or rubbish is left out	O	O	O	O	O	O	O	O	O
8.In cases of severe infestation it may be necessary to cull the animals with traps or poison	O	O	O	O	O	O	O	O	O
9.Government approved rat and mouse poisons can be identified by a number followed by N or NL on the label	O	O	O	O	O	O	O	O	O
**In general, is the public of the opinion that …**	Yes	No	How certain are you? (1 = very unsure – 7 absolutely certain) 1	2	3	4	5	6	7
10.If it is necessary to kill rodents because they are causing a nuisance, this should be done in a humane way	O	O	O	O	O	O	O	O	O
11.Using traps is preferable to using poison because there is less risk of spreading poison in the environment	O	O	O	O	O	O	O	O	O
12.If they were to experience rodent nuisance, they know where to get advice	O	O	O	O	O	O	O	O	O
13.If they were to experience rodent nuisance, they would engage a specialist company	O	O	O	O	O	O	O	O	O
14.If they were to experience rodent nuisance, they would contact the local authority	O	O	O	O	O	O	O	O	O
