# Supplementary material for: What Do They Know? Comparing Public Knowledge and Opinions about Rodent Management to the Expectations of Pest Controllers
Source: Animals (Basel). 2021 Dec 1;11(12):3429. doi: 10.3390/ani11123429 (PMC8698193; doi:10.3390/ani11123429)
Supplement: Supplementary file 1 [file animals-11-03429-s001.zip › S3_Animals 1442441_ Comparisons of female-male and urban-rural responses.pdf]

Supplementary file, S3.

(i) Comparison of results for female vs. male members of the public (n = 314)

Since the number of female respondents was approximately four times the number of male respondents, it is possible that the results were biased. To examine this, the levels of agreement and certainty in the general public were compared for females vs. males. The results can be found in Table S3.1, with numbers in **bold** indicating significant differences (p<0.05, without correction for multiple testing) with chi-squared (for agreement) and t-tests (for certainty).

Table S3.1. Comparison of results for female vs. male members of the public.

Knowledge									
	Q1*	Q2	Q3	Q4	Q5	Q6	Q7	Q8	Q9
**Agreement (n)**									
Female -Yes	242	252	182	201	238	241	227	**201****	177
Female-No	13	3	73	54	17	14	28	**54**	78
Male - Yes	55	57	38	48	53	52	48	**54**	42
Male - No	3	1	20	10	5	6	10	**4**	16
**Certainty (Mean)**									
Females	5,97	6,16	5,24	5,53	5,97	6,26	5,81	5,98	3,22
Males	6,14	6,49	5,3	5,27	6,13	6,48	6,07	5,76	2,96

Opinions					
	Q1	Q2	Q3	Q4	Q5
**Agreement (n)**					
Female -Yes	**223**	204	154	141	152
Female-No	**32**	51	101	114	103
Male - Yes	**43**	46	41	24	37
Male - No	**15**	12	17	34	21
**Certainty (Mean)**					
Females	**5,72**	5,67	5,84	5,41	5,36
Males	**6,2**	5,48	5,58	5,47	4,88

*Q1 – Q9: see Table S3.3 (below) or Table 3 in main article. **Text in **bold**: p<0.05

The number of significant differences between the two sexes were small and the actual differences in response were very small. Females were significantly more likely than males to accept that eradication may be necessary if an infestation was serious. Males were significantly more likely than females to intend to contact the local authority if they experienced rodent nuisance.

(ii) Comparison of results for members of the public living in urban vs. rural areas (n = 314)

It is possible that whether respondents live in an urban or rural area could influence their knowledge and opinions about rodents, so a comparison was carried out between these two groups of respondents. The results can be found in Table S3.2, with numbers in **bold** indicating significant differences (p<0.05, without correction for multiple testing) with chi-squared (for agreement) and t-tests (for certainty).

Table S3.2. Comparison of results for members of the public living in urban vs. rural areas.

Knowledge									
	Q1*	Q2	Q3	Q4	Q5	Q6	Q7	Q8	Q9
**Agreement (n)**									
Urban-Yes	123	125	**98****	98	122	118	111	96	81
Urban-No	4	2	**29**	29	5	9	16	31	46
Rural- Yes	174	184	**122**	151	169	175	164	159	138
Rural - No	12	2	**64**	35	17	11	22	27	48
**Certainty (Mean)**									
Urban	6	6,41	5,2	**5,12**	6,19	6,39	5,93	**5,39**	**2,7**
Rural	6,2	6,47	5,38	**5,47**	6,05	6,49	6,1	**6,09**	**3,22**

Opinions					
	Q1	Q2	Q3	Q4	Q5
**Agreement (n)**					
Urban-Yes	111	101	74	67	79
Urban-No	16	26	53	60	48
Rural- Yes	155	148	121	98	110
Rural - No	31	38	65	88	76
**Certainty (Mean)**					
Urban	6,04	5,28	5,62	**5,26**	4,87
Rural	6,17	5,66	5,63	**5,62**	5,05

*Q1 – Q9: see Table S3.3 (below) or Table 3 in main article. **Text in **bold**: $p<0.05$

There is some evidence for differences in agreement with statements reflecting factual knowledge and opinions. Respondents who lived in the country were significantly more likely to know that sealing cracks in walls would help exclude rodents. They were also more certain that removing plants from walls helped exclude rodents, that eradication may be necessary if an infestation was serious, and that they knew where to get advice.

Table S3.3: The questions posed to participants

**Knowledge**
Q1 Rats and mice belong to the natural wild fauna of the Netherlands.
Q2 Rodents need food, water and shelter to survive.
Q3 You can prevent rodents entering buildings by sealing off cracks in the walls.
Q4 Climbing plants growing up against the exterior walls make it easier for rodents to enter buildings.
Q5 Excluding rodents from buildings is preferential to using traps or poison.
Q6 Food should be stored in containers with tightly fitting lids.
Q7 Rodent infestations can be prevented by not leaving food or rubbish out.
Q8 In serious cases of rodent infestation it may be necessary to eradicate the animals by using traps or poison.
Q9 A rodenticide is approved for use in the Netherlands if it has a N-number or NL-number on the packaging.
**Opinions**
Q1 If I experienced rodent nuisance I would contact the local authority.
Q2 If it is necessary to kill rodents because they have become a pest, this should be done using humane methods.
Q3 The use of traps is preferable to the use of poison because with traps there is no risk of spreading poison in the environment.
Q4 If I experience nuisance from rodents I know where I can get advice.
Q5 If I experience nuisance from rodents, I would contact a specialist company.
